# Implementing dementia risk reduction in primary care: a preliminary conceptual model based on a scoping review of practitioners’ views

**DOI:** 10.1017/S1463423619000744

**Published:** 2019-10-23

**Authors:** Kali Godbee, Jane Gunn, Nicola T Lautenschlager, Eleanor Curran, Victoria J Palmer

**Affiliations:** 1 Department of General Practice, Melbourne Medical School, University of Melbourne, Carlton, Australia; 2 Academic Unit for Psychiatry of Old Age, Department of Psychiatry, Melbourne Medical School, University of Melbourne, Carlton, Australia

**Keywords:** dementia risk reduction, implementation frameworks, primary care, primary prevention, scoping review

## Abstract

Primary care practitioners (PCPs) do not routinely promote dementia risk reduction. The purpose of this study was to map the published literature on the views of PCPs about dementia risk reduction, in order to identify implementation constructs and strategies crucial to the development of an implementation intervention to support dementia risk reduction in primary care. We undertook a scoping review of the PCPs’ views about promoting brain health for reducing dementia risk. We searched MEDLINE, PsycINFO, CINAHL, and Embase for English-language articles published between 1995 and December 2017. We then applied the Consolidated Framework for Implementation Research (CFIR) and matched Expert Recommendations for Implementing Change to the scoping review findings in order to develop a preliminary implementation model. Eight articles reported views of PCPs about dementia prevention. Study findings were mapped to 5 of the 39 CFIR constructs: (i) knowledge and beliefs about dementia risk reduction, (ii) evidence strength and quality, (iii) relative priority, (iv) available resources, and (v) external policy and incentives. The findings suggest implementation strategies to consider in our preliminary model include (i) educational meetings, (ii) identifying and preparing champions, (iii) conducting local consensus discussions, (iv) altering incentive structures, and (v) capturing and sharing local knowledge. There have been few studies about the views of PCPs about dementia risk reduction. Implementation in the primary care setting is fundamental to early identification of risk and supporting preventive practices, but it needs to focus on more than just education for PCPs. We need more up-to-date and in-depth data on the views of PCPs about dementia risk reduction and context-specific analyses of implementation needs. Further research into effective primary care interventions to reduce dementia risk is expected to support implementation efforts.

## Introduction

Dementia affects over 46 million people worldwide, and this figure is expected to triple over the next 30 years (Prince, 2015). In the absence of a cure for dementia, prevention has become ‘the Holy Grail’ (Bassil and Grossberg, 2009:29), and there has been growing interest from researchers, policy-makers, and the public in reducing dementia risk (Livingston *et al*., 2017).

Epidemiological evidence suggests that up to one-third of all dementia cases are attributable to modifiable risk factors such as obesity, hypertension, diabetes, physical inactivity, and smoking (Livingston *et al*., 2017). There is not yet strong evidence for specific interventions that can reduce dementia risk (Kane *et al*., 2017), although multi-domain lifestyle interventions are currently being trialled in the USA, Europe, Singapore, and Australia (Kivipelto, 2018). While we await the results of these trials, experts agree there is sufficient evidence for the provision of general advice and support to manage likely risk factors for dementia. Such advice and support might have specific benefits for reducing dementia risk, are unlikely to do harm, and can have broader health benefits (Public Health England, 2014; Smith and Yaffe, 2014; National Academies of Sciences, 2017).

Primary care could be a suitable setting for the provision of this advice and support (RACGP, 2006; Travers *et al*., 2009). No other health care system has the population reach of primary care. About half of the middle-aged patients in primary care have at least one modifiable risk factor for dementia (Fryar *et al*., 2012; Australian Bureau of Statistics, 2015; British Heart Foundation, 2018), and they see a primary care practitioner (PCP) three times a year in the USA (National Centre for Health Statistics, 2015) and six times a year in Australia (Britt *et al*., 2016b) and England (British Medical Association, 2017), often for minor, self-limiting problems (RCGP, 2007). Key policy and guideline documents from Australia (RACGP, 2016), the UK (NICE, 2015), and the USA (American Academy of Family Physicians, 2019) specify a role for PCPs in the promotion of brain health for the possible reduction of dementia risk. This role includes educating patients that dementia is not a normal part of ageing and supporting patients to improve heart health, exercise, eat a healthy diet, increase mental stimulation, and increase social activity.

Simple though this advice and support may seem, it is not routinely delivered in primary care. Among 4728 respondents to a 2009 consumer survey in the USA, only 7.8% of consumers said a PCP had talked with them in the past 12 months about ways to stay mentally sharp (Friedman *et al*., 2013). In Australia and the UK, only 15% of 621 patients surveyed said they had heard about dementia from their general practitioner (Millard *et al*., 2011). In fact, PCPs only occasionally deliver any preventive advice and support (Rafferty, 1998; McDonnell *et al*., 2001; Hung *et al*., 2007; Joyce and Piterman 2011; Britt *et al*., 2016a), let alone advice and support specific to reducing dementia risk. This means that, even though many of the risk factors for dementia overlap with the risk factors for other chronic diseases, it is unlikely that dementia risk is being managed incidentally.

There is a need to bridge the gap between current practice and the routine promotion of brain health for dementia risk reduction. Implementation science is a relatively new area of health research that focusses on bridging such gaps in practice, with implementation models used to describe the process of changing routine practice (Nilsen, 2015). Implementation models offer practical guidance in the planning and execution of strategies by elucidating important aspects to be considered in implementation practice. Critically, implementation models in primary care are multi-level, focussing not just on the PCP but also on the organisation and broader health system (Holtrop *et al*., 2018).

The first step in developing an implementation model for dementia risk reduction in primary care is to identify the reasons why PCPs do not yet routinely promote brain health. Currently, these reasons are not well understood. Some clues can be deduced from the broader preventive literature. Systematic reviews focussing on primary care prevention of diabetes (Messina *et al*., 2017), prevention of any cardiometabolic disease (Wändell *et al*., 2018), or reducing lifestyle risk factors in more general terms (Stead *et al*., 2009; Eisner *et al*., 2011; Taylor *et al*., 2011; Rubio-Valera *et al*., 2014) have highlighted common challenges. These include (but are not limited to) lack of knowledge, skills, or resources for primary prevention and health promotion behaviours, as well as a system-wide focus on treatment that leaves little time or reimbursement for prevention. Given the overlap in modifiable risk factors, it is likely that similar barriers exist for preventing dementia as for preventing diabetes and other cardiometabolic diseases. However, providing advice and support to reduce dementia risk factors also carries unique salient concerns, given the lack of evidence for specific interventions that reduce dementia risk, the stigma associated with dementia, and its typical onset in later life (Mitchell *et al*., 2017).

To develop an implementation model for dementia risk reduction in primary care, we first need to understand how PCPs view dementia risk reduction and their role in promoting brain health, and the barriers and facilitators they perceive to embedding it in routine practice.

This study sought to answer two research questions:

1. What is the nature of published evidence on the views of PCPs about promoting brain health for dementia risk reduction?
2. Which implementation strategies could address the barriers to dementia risk reduction identified in the scoping review?

## Methods

This article reports the study in two parts: a scoping review, and then an application of the Consolidated Framework for Implementation Research (CFIR) and matched Expert Recommendations for Implementing Change (ERIC) to the findings of the scoping review in order to develop a preliminary implementation model.

Scoping reviews are useful for answering broad questions such as ‘What is known about a concept?’ and for examining emerging evidence when it is still unclear what other, more specific questions can be posed and valuably addressed by other research methods (Peters *et al*., 2015; Tricco *et al*., 2016). We followed guidance from the Joanna Briggs Institute for conducting systematic scoping reviews (Peters *et al*., 2015; Peters *et al*., 2017). The review is reported in accordance with the PRISMA Extension for Scoping Reviews (PRISMA-ScR) Checklist (Tricco *et al*., 2018).

### Scoping review eligibility criteria

The aim of the scoping review was to identify the current literature on PCPs’ views about dementia risk reduction and promoting brain health. Only studies that focussed on PCPs were eligible for inclusion. Our definition of PCPs included General Practitioners, General Practice Registrars, Nurse Practitioners, General Practice Nurses, Physician Assistants, and any international equivalents; allied health professionals were not included. Studies that comprised mixed samples of PCPs and other health professionals were included if PCP data were presented separately and could be considered independent of other findings or if PCPs comprised at least half of the mixed sample.

We included quantitative, qualitative, and mixed-method studies with data pertaining to PCPs’ views, attitudes, or beliefs about dementia prevention, dementia risk reduction, or maintaining cognitive and brain health. We excluded studies that only reported participants’ knowledge or understanding of dementia as a disease (i.e., health literacy) and studies that were only concerned with dementia screening, diagnosis, or management.

To ensure minimum standards for quality and reporting, only published journal articles were included. We included articles published between 1995 and 2018 and written in English. Articles published before 1995 predate significant advances in the scientific understanding of dementia prevention, and we were not resourced to translate studies in languages other than English.

### Search strategy and study selection

The strategy was designed with the assistance of a librarian and executed by KG. On 18 December 2018, we searched MEDLINE, PsycINFO, CINAHL, and Embase with predetermined date and language restrictions using terms based on ‘dementia’, ‘prevention’, and ‘views’, ‘attitudes’, or ‘beliefs’ (Supplementary File 1 details the complete search strategy for each database). We did not specifically search for studies that focussed on PCPs, partly because the terms for PCPs vary widely across health care systems but also because we were interested to compare the number of studies focussing on PCPs with the number of studies concerned with the views of laypersons and other populations.

Duplicate references were deleted. Two reviewers (KG and JS) screened titles and abstracts of a randomly selected subsample of 10% of unique records, discussed the results, and amended the screening and data extraction manual. KG screened the titles and abstracts of the remaining records. KG obtained the full text of potentially eligible articles, of which 20% were assessed by two reviewers (KG and EC). Any discordance or uncertainty was resolved through discussion between the two reviewers initially and the involvement of a third reviewer (VP) as necessary. The remaining records were evaluated for inclusion by KG. The electronic database search was supplemented by searching the reference lists of included articles and searching Google Scholar for references citing the included articles; one additional record was included from the supplemental search.

### Data charting

A manual data-charting form was jointly developed by KG and VP. We extracted data on country of study, year of data collection, study aim, design, participants, and relevant results. A formal assessment of methodological quality of included studies was considered incongruent with the aim of the scoping review, which was to provide an overview of the existing evidence regardless of quality (Peters *et al*., 2015). Data were charted by KG and verified by VP for all included studies. Results were synthesised into a table ordered by study design (qualitative first), then country of study (most frequent country first), and then year of data collection (oldest first).

### Applying the CFIR

We applied the CFIR (Damschroder *et al*., 2009) to the findings of the scoping review. Determinant frameworks such as the CFIR describe general domains of barriers and facilitators that influence implementation (Nilsen, 2015). This is critical to consider in changing any professional practice and for consideration of systems change. The CFIR is one of the most commonly used determinant frameworks (Birken *et al*., 2017) and is based on theories identified in Greenhalgh et al.’s (2004) widely cited systematic review. The CFIR is composed of 39 constructs in 5 major domains. In the context of dementia risk reduction, the intervention characteristics domain refers to issues such as the complexity or cost of promoting brain health. The characteristics of individuals domain refers to attributes of individual PCPs, such as their confidence and beliefs about promoting brain health. The inner setting domain refers to the primary care organisation, including its culture and readiness for implementation. The outer setting domain refers to constructs beyond the primary care organisation, such as patient needs and external policies. Finally, the implementation process domain incorporates strategies and tactics that might influence implementation, such as forward planning and engaging appropriate individuals.

To develop our preliminary implementation model, we mapped each of the relevant findings from the scoping review to 1 of the 39 CFIR constructs.

### Applying the CFIR–ERIC Matching Tool

In 2015, the ERIC project systematically gathered input from a wide range of stakeholders with expertise in implementation science and clinical practice in order to compile a list of 73 clearly defined implementation strategies (Powell *et al*., 2015). In a separate study, Damschroder *et al*. (2015) invited implementation experts to select and rank up to 7 ERIC strategies they thought would best address each of the 39 constructs in the CFIR, which they had translated into barriers. From this work, the CFIR Research Team developed the CFIR–ERIC Matching Tool v1.0 (CFIR Research Team, 2018).

The CFIR–ERIC Matching Tool works by inviting the user to enter relevant CFIR barriers, then returning the list of 73 ERIC implementation strategies sorted from strongest to weakest according to the strength of endorsement for the combination of the CFIR constructs the user entered. The tool is designed to guide selection of implementation strategies with a high priority for consideration in designing an implementation intervention (CFIR Research Team, 2018).

We entered the CFIR constructs we identified in the scoping review into the CFIR–ERIC Matching Tool and selected the top five ERIC strategies for inclusion in our preliminary implementation model.

## Results

**Figure 1.**
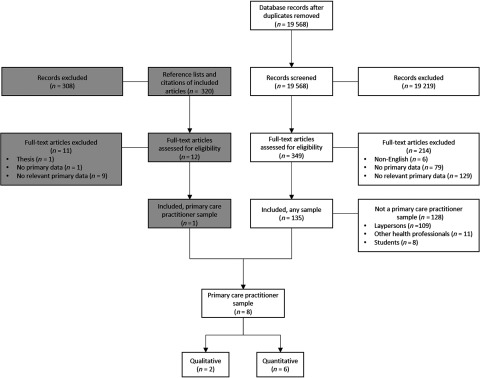
Flow diagram of evidence selection

The flow diagram in Figure 1 summarises the process of evidence selection. Inter-rater reliability for determining inclusion of full-text articles was 96%. All excluded full-text papers are listed in Supplementary File 2 with reasons for exclusion. In total, eight articles reported views, attitudes, or beliefs of PCPs about dementia prevention. Two articles reported data from the same qualitative study, for a total of seven studies included in this review.

Table 1 charts the extracted characteristics of the seven included studies. Data were available for 3368 participants, of whom 3006 (89%) were PCPs. Two studies included specialists in the sample (Chase *et al*., 2002; Wilkinson *et al*., 2005). The newest data were 8 years old, with data from most studies collected over 10 years ago.

**Table 1.** Characteristics of included studies

Authors	Country	Year of data collection	Aim	Design	Participants	PCPs (n)	Relevant results
Hochhalter *et al*. (2012); Warren-Findlow *et al*. (2010)	United States	2007/2008	To describe primary care providers’ perceptions and practices regarding cognitive health, their preferred sources of information on cognitive health, and their perceptions of the best ways to disseminate cognitive health information	10 focus groups and 3 interviews	28 general practitioners (57%) 21 physician assistants and nurse practitioners (43%)	49	Some participants felt evidence on the efficacy of preventive strategies for cognitive health was insufficient Many participants reported suggesting disease management and activities such as games and social interaction PCPs identified barriers to talking with patients about cognitive health lack of time patient reactions to recommendations only the patient’s most urgent concerns can be addressed discontinuity of care across health care settings and providers
Chase *et al*. (2002)	United States	2002	To examine physician knowledge, preferences, and use of genetic tests for AD	Postal survey	general internists or family practitioners (62%) specialists (38%)	106	Substances recommended for the possible prevention of memory loss^†^ Aricept (74.3%) oestrogen (60.2%) vitamin E (59.6%) ginkgo (34.5%) Cognex (27.5%) nonsteroidal anti-inflammatory medications (24.6%) vitamin C (18.7%)
Day *et al*. (2012)	United States	2008	To examine primary care physicians’ perceptions and self-reported practices related to reducing the risk of cognitive impairment or dementia	Web-based survey	493 family or general practitioners (51%) 479 internists (49%)	972	Types of advice^†^ physical activity (91.8%) intellectual stimulation (85.3%) healthy diet (83.2%) social activity (79.7%) limiting alcohol (64.4%) maintaining a healthy weight (52.6%) nutritional supplements (34.2%) preventive medications (16.9%) Strength of evidence for reducing risk of cognitive impairment Weak or very weak (39.2%) Moderate, strong, or very strong (54.7%) Cannot be determined from current scientific evidence (6.1%) Biggest barrier to addressing risk of cognitive impairment lack of reimbursement and time (31.9%) limited scientific evidence or proven treatments (26.3%), patients’ more immediate health issues (24.6%) patients unlikely to comply (9.5%) no major barriers (7.7%)
Friedman *et al*. (2013)	United States	2009	To compare the perceptions of consumers and primary care providers related to beliefs and communication practices about lifestyle behaviours beneficial for overall health and for maintaining cognitive functioning	Web-based survey	1000 family physicians, general practice physicians, or internists (80%) 250 nurse practitioners (20%)	1250	Types of advice (physicians; nurse practitioners)^†^ physical activity (86.1%; 85.2%) intellectual stimulation (80.2%; 80.8%) healthy diet (60.9%; 65.2%) social activity (66.7%; 70.0%) limiting alcohol (59.1%; 57.2%) maintaining a healthy weight (45.7%; 42.8%) nutritional supplements (29.3%; 36.4%) preventive medications (11.6%; 10.0%)
Wilkinson *et al*. (2005)	France, Germany, Italy, Poland, Spain, and the UK	2004	To assess the awareness of and behaviours surrounding AD and dementia among all key stakeholders in Europe^‡^	Telephone or face-to-face survey	308 general practitioners or primary care physicians (51%) 297 specialists (49%)	308	Belief that age does not necessarily lead to deteriorating memory (56%) Belief that age does not necessarily affect one’s ability to express oneself (54%)
Werner *et al*. (2013)	Israel	2011	To explore the familiarity, knowledge, help-seeking, and treatment preferences of family physicians regarding MCI	Self-administered survey distributed during professional meetings and in continuing education courses	197 family physicians	168	Family physicians’ preferences for treatment for a person with MCI^†^ Relaxation exercises (46.6%) Yoga or meditation (34.4%) Pharmacological treatment (13.0%) Engagement in social activities (87.5%) Natural medications (9.6%) Vitamins (33.8%) Engagement in physical activity (88.1%) Cognitive training (88.2%) Change in diet (44.0%)
Tang *et al*. (2016)	England	2014	To evaluate the current attitudes and experiences of future GPs in dementia care and their views on targeting high-risk groups	Emailed survey	153 GP trainees	153	Primary care has a key role to play in identifying those at high risk of dementia (90.4%) A risk prediction tool would be helpful to identify those at high risk of dementia (61.1%)

† Advice categories were not mutually exclusive; participants could select more than one option.

‡ The aim is inferred from Bond (2005), to which Wilkinson *et al*. (2005) refer the reader for ‘a more complete description of survey methodology and participants’ (p. 28).

AD = Alzheimer’s disease; MCI = Mild cognitive impairment.

### Findings from the scoping review, mapped to CFIR constructs

Collectively, the studies identified in the scoping review examined several aspects of participants’ views on dementia risk reduction. We were able to map these findings to 5 of the 39 CFIR constructs: (i) knowledge and beliefs about the intervention (in the ‘Characteristics of Individuals’ domain), (ii) evidence strength and quality (in the ‘Intervention Characteristics’ domain), (iii) relative priority (in the ‘Inner Setting’ domain), (iv) available resources (in the ‘Inner Setting’ domain), and (v) external policy and incentives (in the ‘Outer Setting’ domain). This application of the CFIR provided the basis for the later selection of relevant implementation strategies to support the promotion of dementia risk reduction in the primary care setting.

The findings from the scoping review are mapped to the five relevant CFIR constructs below.

1. Knowledge and beliefs about the intervention

All seven studies reported data related to the individual attitudes of PCPs towards promoting brain health, the value they place on promoting brain health, or their familiarity with facts, truths, and principles related to promoting brain health.

Just over half of the PCPs in the 2004 European survey believed that age does not necessarily lead to deteriorating memory or affect one’s ability to express oneself (Wilkinson *et al*., 2005). Over 90% of GP trainees agreed that primary care had a key role to play in identifying those at high risk of dementia (Tang *et al*., 2016).

In terms of their attitude and approach to promoting brain health, most PCPs in the 2008 and 2009 US surveys and the 2011 Israeli survey recommended physical activity, intellectual stimulation, and social activity for dementia risk reduction rather than nutritional supplements, vitamins, and preventive medications. More than half of the respondents to the 2002 survey recommended nutritional supplements and vitamins and preventive medications, but this advice was less commonly provided in later studies. About half of the PCPs in the 2008 and 2009 US surveys advised patients to limit alcohol intake and maintain a healthy weight to reduce dementia risk (Day *et al*., 2012; Friedman *et al*., 2013); the other survey studies did not examine respondents’ attitudes to advice on alcohol or weight.

In the qualitative study, participants cited a range of recommendations to promote brain health, including staying busy, volunteering, being socially engaged, being physically active, eating healthfully, doing puzzles or other games, reading, and learning new things or trying new activities. PCPs in the qualitative study also stressed disease management including controlling cholesterol, hypertension, diabetes, and other medical conditions (Hochhalter *et al*., 2012).

In summary, the scoping review indicated beliefs and attitudes towards promoting brain health were generally favourable for implementation in primary care, although some participants were unsure whether dementia was preventable and which risk factors to target.

1. *Evidence strength and quality*

Two studies examined PCPs’ perceptions of the quality and validity of evidence supporting the belief that promoting brain health will have desired outcomes. In the qualitative study, some PCPs expressed concern that current cognitive health research is inconclusive (Warren-Findlow *et al*., 2010), whereas others felt there was no harm in making recommendations based on weak evidence (Hochhalter *et al*., 2012). Some participants felt that vascular dementia was preventable, whereas Alzheimer’s disease was not (Hochhalter *et al*., 2012).

In the 2008 US survey, over one-third of the physicians believed evidence for reducing dementia risk was weak, and a quarter of the respondents felt the limited scientific evidence and the lack of proven treatments were the biggest barriers to discussing dementia risk reduction with their patients (Day *et al*., 2012). Not only did some PCPs perceive there to be limited evidence that dementia risk can be reduced, there was a sense that patients were unlikely to adhere to brain health promotion advice anyway. For 9.5% of PCPs in the 2008 survey, anticipated non-adherence to advice was the biggest perceived barrier to discussing dementia prevention (Day *et al*., 2012).

1. *Relative priority*

Two studies considered the relative priority of promoting brain health in the primary care setting. In the 2008 US survey, 24.6% of the PCPs felt patients’ more immediate health issues were the biggest barriers to discussing dementia prevention with patients (Day *et al*., 2012). Participants in the qualitative study similarly felt that only patients’ most urgent concerns could be addressed, which did not include discussions on brain health, about which participants felt few patients were concerned (Hochhalter *et al*., 2012).

1. *Available resources*

Three studies considered the impact of available resources for promoting brain health. In the 2008 US survey, 31.9% of the PCPs viewed lack of time and reimbursement as the biggest barriers to discussing dementia prevention with patients (this finding was also mapped to external policies and incentives, below) (Day *et al*., 2012). Time constraints were ‘almost universally acknowledged’ by participants in the qualitative study to be a barrier to discussions about cognitive health (Hochhalter *et al*., 2012, p. 4).

In the GP trainee study, additional resources were considered important. Specifically, three fifths of GP trainees (61.1%) thought a risk prediction tool would be helpful to identify those at high risk of dementia (Tang *et al*., 2016).

1. *External policy and incentives*

Two studies considered the impact of policy and incentives outside the individual primary care organisation, albeit indirectly. As mentioned above, 31.9% of the PCPs in the 2008 US survey viewed lack of reimbursement (and time) as the biggest barriers to discussing dementia prevention with patients (Day *et al*., 2012). Separate to the issue of reimbursement, participants in the qualitative study viewed discontinuity of care across health care settings and providers as a barrier to developing relationships needed to discuss sensitive topics like brain health (Hochhalter *et al*., 2012).

### Development of a preliminary implementation model, using the CFIR–ERIC Matching Tool

**Figure 2.**
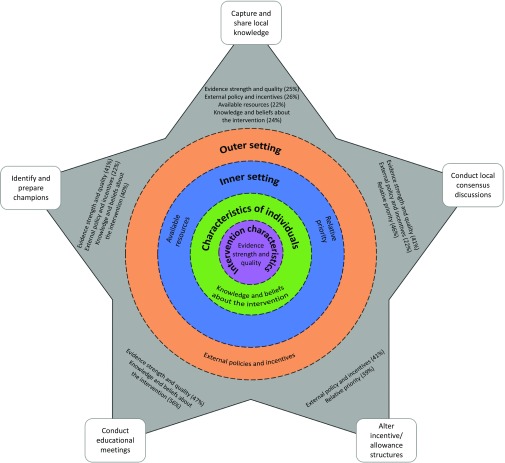
Preliminary implementation model for dementia risk reduction in primary care

Figure 2 depicts our preliminary implementation model for dementia risk reduction in primary care. The five relevant CFIR constructs are shown within their domains, which radiate in concentric circles from intervention characteristics at the centre to the outer setting. Using the CFIR–ERIC Matching Tool, we identified the five strategies with the strongest relative strength of endorsement for the combination of the five relevant CFIR constructs. These implementation strategies were to (i) identify and prepare champions, (ii) conduct educational meetings, (iii) conduct local consensus discussions, (iv) alter incentive/allowance structures, and (v) capture and share local knowledge.

These five implementation strategies are shown as points of a grey star surrounding the CFIR constructs in their domains. For all matches endorsed by at least 20% of implementation experts, the proportion of implementation experts endorsing the strategy as a match for each construct is listed beside the strategy in Figure 2.

An implementation intervention to support the promotion of brain health in the primary care setting should consider identifying and preparing ‘champions’: individuals who will dedicate themselves to supporting, marketing, and driving through the promotion of brain health. Champions may help shift perceptions about the preventability of dementia, and they can reinforce the message from experts that we cannot afford to wait for more robust evidence for interventions to reduce dementia risk.

Educational meetings are also recommended as an implementation strategy. Teaching PCPs how to promote dementia risk reduction in primary care should improve their approach to giving advice and lead to more positive beliefs about implementation.

The third implementation strategy to consider is including local PCPs and patients in consensus discussions about the importance of dementia risk reduction and the appropriateness of PCPs promoting brain health with at-risk patients. This should help raise the relative priority of brain health promotion in the primary care organisation and improve the perception that it could reduce dementia risk.

Altering incentive or allowance structures could address the current lack of reimbursement for promoting brain health and ensure that brain health promotion is seen as ‘real work’ by PCPs. Altered structures could also incentivise patients to see their ‘usual’ doctor, maintaining continuity of care.

Finally, capturing and sharing local knowledge from successful implementers on how they have made brain health promotion ‘work’ in their setting is another strategy that could help overcome the barriers to promoting brain health identified in the scoping review (although it is unlikely to raise the priority relative to patients’ more immediate health issues).

## Discussion

This study set out to characterise the nature of published evidence on PCPs’ views about promoting brain health for dementia risk reduction. Only 7 studies were identified that examined PCPs’ views about promoting brain health, in contrast to over 100 studies pertaining to the views of laypersons. The limited data suggest that PCPs generally had positive beliefs and attitudes towards promoting brain health, but there was some uncertainty whether dementia was preventable and which risk factors to target. Some participants perceived there to be poor evidence supporting dementia risk reduction, and the promotion of brain health was viewed as a low priority relative to patients’ more immediate health issues. In terms of resources, PCPs wanted more time to promote brain health, and some also wanted a risk prediction tool to help identify at-risk patients. External policies and incentives regarding reimbursement and continuity of care were seen to undermine efforts to implement brain health promotion.

The dementia-specific findings in the current review are consistent with findings from earlier systematic reviews of the barriers and enablers of primary prevention and health promotion in primary care more broadly. For example, in the prevention of cardiometabolic diseases in primary care, barriers to preventive care have included negative attitudes to prevention, time restraints, ineffective interventions, and insufficient reimbursement, whereas facilitators were a sense of responsibility to offer prevention and its importance (Wändell *et al*., 2018). Diabetes prevention activities in primary care have likewise been impacted by PCPs’ workload, time constraints, resources, and self-efficacy, and their perception of patient motivations towards change (Messina *et al*., 2017). It appears these barriers are entrenched in primary care, reinforcing the need for an implementation model to guide the process of changing routine practice. Simply providing PCPs with guidelines for dementia risk reduction will not be enough to change their professional behaviour (Cabana *et al*., 1999). Underlying organisational realities, beyond the domain of individual PCPs, need to be considered (Checkland *et al*., 2007).

This study also set out to develop a preliminary model for the implementation of dementia risk reduction based on the findings from the scoping review. The barriers to the promotion of brain health identified in the current review are likely to be addressed by an implementation model that incorporates matching implementation strategies. According to the CFIR–ERIC Matching Tool, matched strategies include identifying and preparing champions, conducting educational meetings, conducting local consensus discussions, altering incentive structures, and capturing and sharing local knowledge.

It might not be necessary to incorporate all these strategies to achieve successful implementation. In their systematic review of reviews on achieving change in primary care, Lau et al. (2015) found that multifaceted interventions were not necessarily more effective than single strategies alone. This is particularly the case when baseline adherence to desired practice is low, as with promoting brain health for dementia risk reduction. The most effective approach to implementing dementia risk reduction will likely involve working with PCPs and patients in individual primary care organisations to prioritise and adapt strategies according to their specific needs and resources (Meyers *et al*., 2012).

Whichever implementation approach is taken, the findings of the scoping review suggest it should not rely on educational meetings alone. Typically, key policy and guideline documents have focussed on training and continuing professional development programs for PCPs to promote brain health, including how to identify people at most risk and how to advise and support people to change behaviour (e.g., NICE, 2015). However, the findings of this scoping review suggest that most PCPs already have positive beliefs and attitudes about dementia risk reduction. Furthermore, the experts contributing to the development of the CFIR–ERIC Matching Tool agree that educational meetings are unlikely to address other relevant barriers such as the relative low priority of brain health promotion within the primary care setting or the lack of time and the lack of risk assessment tools, reimbursement, and continuity of care (CFIR Research Team, 2018). An implementation intervention that focusses on education and training alone is unlikely to be maximally effective in supporting the promotion of brain health in primary care (Forsetlund *et al*., 2009).

There are some important caveats to interpreting the findings of this study. A registered *a priori* protocol specifying greater involvement of independent reviewers would have strengthened the methodology, and there may have been relevant evidence in the excluded grey literature. Only 5 of the 39 constructs of the CFIR could be identified in the findings from the scoping review. Given that most of the studies used closed-ended surveys, it is possible that the narrow range of identified constructs reflected the narrow scope of the survey questions. Several of the studies were also quite dated and PCPs’ views might have shifted with the emergence of new evidence about dementia risk factors.

The extent to which the expert endorsements underpinning the CFIR–ERIC Matching Tool apply to the promotion of brain health in the primary care setting is an open question (CFIR Research Team, 2018). The matching tool may help inform implementation planning efforts, but it is not a substitute for a local, context-specific analysis of implementation needs. For example, US participants in the qualitative study felt that discontinuity of care was a barrier to dementia risk reduction in primary care. This barrier might not be evident in the UK with its patient registration system (Jones, 2018) or in Australia where 86% of Australians aged 45 and over have a usual GP and 74% of those can always see their preferred GP when needed (Australian Bureau of Statistics, 2018). There may be little added value in altering incentive structures in the UK or Australia to encourage continuity of care. Similarly, educational meetings were endorsed by 47% of experts as one of the top seven strategies to address negative perceptions of the quality and validity of evidence supporting an intervention. Education might help when there is simply low awareness of robust evidence, but it may be of limited value where negative perceptions of the evidence base are well-founded, as is the case for specific interventions to reduce dementia risk. Improving perceptions of the evidence for dementia risk reduction might depend more on building the evidence base than on educational meetings.

The literature-based implementation model presented here is necessarily preliminary; it has yet to be tested and refined. We aim to refine the model for the Australian context by collecting more up-to-date and nuanced data on the views of Australian PCPs about dementia risk reduction. Following principles of patient and public involvement (Robert *et al*., 2015), we also aim to bring together groups of PCPs and patients working within the same general practice to share their experiences of brain health promotion, identify their shared priorities for implementation, and then work on the identified priorities. Evaluation of the codesigned implementation process, incorporating both observation and explanatory narratives offered by participants (Checkland *et al*., 2007), will be used to further refine the implementation model.

To our knowledge, this is the first published study using the CFIR–ERIC Matching Tool to select implementation strategies based on determinants of behaviour, and one of the first scoping reviews reported in accordance with the new PRISMA-ScR Checklist (Tricco *et al*., 2018). This review has captured PCPs’ views about constructs important to the development of an implementation intervention to support dementia risk reduction in primary care.

## Supplementary material

For supplementary material accompanying this paper visit https://doi.org/10.1017/S1463423619000744. click here to view supplementary material

## Prior presentations

Preliminary findings from this study were presented as a poster at the NNIDR Australian Dementia Forum and the International Dementia Conference, Sydney, Australia, June 2018, and at the Primary Health Care Research Conference, Melbourne, Australia, August 2018.
