# Supplementary material for: Implementing dementia risk reduction in primary care: a preliminary conceptual model based on a scoping review of practitioners’ views
Source: Prim Health Care Res Dev. 2019 Oct 23;20:e140. doi: 10.1017/S1463423619000744 (PMC6842648; doi:10.1017/S1463423619000744)
Supplement: Supplementary file 1 [file S1463423619000744sup.zip › S1463423619000744sup001.docx]

**Supplementary File 1: Complete search strategy for each database**

All electronic database searches were performed on December 18, 2018.

Medline (Ovid): 9,930 citations

1. (Alzheimer* or Cogn* decline or Cogn* impair* or dementia or Impair* cogn* or Memory complain* or Memory loss or predementia).mp.

2. (onset or prevent* or protect* or risk*).mp.

3. (attitude* or barrier* or belief* or enable* or experience* or facilitat* or focus group* or interview* or needs or opinion* or perspective* or qualitative or themes or view*).mp.

4. 1 and 2 and 3

5. limit 4 to (english language and humans and yr="1995 -Current")

[mp=title, abstract, original title, name of substance word, subject heading word, keyword heading word, protocol supplementary concept word, rare disease supplementary concept word, unique identifier, synonyms]

PsycINFO (Ovid): 7,423 citations

1. (Alzheimer* or Cogn* decline or Cogn* impair* or dementia or Impair* cogn* or Memory complain* or Memory loss or predementia).mp.

2. (onset or prevent* or protect* or risk*).mp.

3. (attitude* or barrier* or belief* or enable* or experience* or facilitat* or focus group* or interview* or needs or opinion* or perspective* or qualitative or themes or view*).mp.

4. 1 and 2 and 3

5. limit 4 to (human and english language and yr="1995 -Current")

[mp=title, abstract, heading word, table of contents, key concepts, original title, tests & measures]

Embase (Ovid): 8,061 citations

1. (Alzheimer* or Cogn* decline or Cogn* impair* or dementia or Impair* cogn* or Memory complain* or Memory loss or predementia).ti.

2. (onset or prevent* or protect* or risk*).mp.

3. (attitude* or barrier* or belief* or enable* or experience* or facilitat* or focus group* or interview* or needs or opinion* or perspective* or qualitative or themes or view*).mp.

4. 1 and 2 and 3

5. limit 4 to (human and english language and yr="1995 -Current")

[ti=title]

[mp=title, abstract, heading word, drug trade name, original title, device manufacturer, drug manufacturer, device trade name, keyword, floating subheading word]

CINAHL: 4,223 citations

1. Alzheimer* or "Cogn* decline" or "Cogn* impair*" or dementia or "Impair* cogn*" or "Memory complain*" or "Memory loss" or predementia

2. onset or prevent* or protect* or risk*

3. attitude* or barrier* or belief* or enable* or experience* or facilitat* or “focus group*” or interview* or needs or opinion* or perspective* or qualitative or themes or view*

4. 1 and 2 and 3

5. limit 4 to (human and English Language and published date “19950101-20181231”)
