# Supplementary material for: Implementing dementia risk reduction in primary care: a preliminary conceptual model based on a scoping review of practitioners’ views
Source: Prim Health Care Res Dev. 2019 Oct 23;20:e140. doi: 10.1017/S1463423619000744 (PMC6842648; doi:10.1017/S1463423619000744)
Supplement: Supplementary file 1 [file S1463423619000744sup.zip › S1463423619000744sup003.docx]

**Supplementary File 2: Excluded full-text papers with reasons for exclusion**

Part A: Database search (n=214 articles excluded)

*Reason 1: Non-English (n=6)*

| **Rec#** | **Author (Year)** | **Title** | **Journal** |
| --- | --- | --- | --- |
| 1 | Broche-Perez, Y., Z. Fernandez-Fleites, B. Gonzalez, M. A. Hernandez Perez and Y. I. Salazar-Guerra (2018). | Knowledge and beliefs about dementia among the general public: A preliminary report on the Cuban population. | Neurologia, no pagination. |
| 2 | Okuno, J., T. Fukasaku, K. Hotta, N. Yabushita, L. Pei, T. Okura, K. Tanaka and H. Yanagi (2013). | Evaluation of the association between cognitive impairment and the vitamin D levels among community-dwelling Japanese pre-frail elderly individuals. | Japanese Journal of Geriatrics 50(4): 515-521. |
| 3 | Pakzad, S., J. Jbilou, M.-C. Paulin, V. Fontaine, D. Donovan, M. Bélanger and P.-É. Bourque (2013). | Accès au diagnostic précoce de démence au Nouveau-Brunswick : perceptions d'usagers potentiels de services selon la langue et le milieu de vie. | Canadian Journal of Public Health 104(S1): S16-20. |
| 4 | Penaherrera-Oviedo, C. A., D. Moreno-Zambrano, M. C. Duarte-Martinez, M. B. Jurado, R. Santibanez, L. Tamariz and A. Palacio (2014). | Concept of dementia as a disease in a population group in the city of Guayaquil. | Revista Ecuatoriana de Neurologia 23(1-3): 29-33. |
| 5 | Serrano, C. M., F. A. Filippin and K. Eiguchi (2016). | Survey about ethics and research in prodromal Alzheimer disease. | Neurologia Argentina 8(4): 237-245. |
| 6 | Urakami, K. (2011). | Examination and preventive intervention of dementia from the point of view of life style. | Japanese Journal of Geriatrics 48(2): 118-119. |

*Reason 2: No primary data (n=79)*

| 1 | Abley, C. (2010). | Dementia research. | Nursing Older People 22(3): 14. |
| --- | --- | --- | --- |
| 2 | Allen, D. (2008). | Growing attention to dementia gives hope. | Nursing Older people 20(3): 12. |
| 3 | Andrieu, S., N. Coley, V. Gardette, J. Subra, S. Oustric, T. Fournier, J. P. Poulain, D. Coniasse-Brioude, V. Igier, B. Vellas and A. Grand (2012). | Representations and practices of prevention in elderly populations: Investigating acceptance to participate in and adhesion to an intervention study for the prevention of Alzheimer's disease (accept study)-The need for a multidisciplinary approach. | Journal of Nutrition, Health and Aging 16(4): 352-354. |
| 4 | Anonymous (2009). | Alzheimer Research Forum Live Discussion: sports concussions, dementia, and ApoE genotyping: what can scientists tell the public? What's up for research? | Journal of Alzheimer's Disease 16(3): 657-666. |
| 5 | Anonymous (2009). | Your genetic makeup is an Alzheimer's risk predictor. If you know you carry a gene that boosts your risk of AD, you may make better decisions about your future. | DukeMedicine healthnews 15(10): 4-5. |
| 6 | Anonymous (2010). | Can modifiable heart and health factors affect Alzheimer's risk? Some risk factors have been shown to protect against cognitive decline. | Heart advisor / the Cleveland Clinic 13(9): 5. |
| 7 | Anonymous (2012). | Will ginkgo biloba keep me from developing dementia? | The Johns Hopkins medical letter health after 50 23(12): 7. |
| 8 | Anonymous (2013). | First rule of dementia prevention: take care of your heart. What you need to know about the link between cognitive decline, Alzheimer's and clogged arteries. | Harvard heart letter : from Harvard Medical School 24(1): 5. |
| 9 | Anonymous (2013). | The road map to integrated dementia prevention and care. | The Lancet Neurology 12(9): 839. |
| 10 | Aschwanden, C. (2004). | Taking the long view. | Science of Aging Knowledge Environment 2004(26): ns3. |
| 11 | Beatty, G. E. (2006). | Shedding light on Alzheimer's. | The Nurse practitioner 31(9): 32-43; quiz 43-45. |
| 12 | Berry, L. (2009). | The National Dementia Strategy: transforming care. | Nursing older people 21(1): 3. |
| 13 | Brown, A. and P. Roach (2010). | My husband has young-onset dementia: A daughter, wife and mother's story. | Dementia 9(4): 451-453. |
| 14 | Buri, H. (1999). | Focus on Research. | British Journal of Occupational Therapy 62(12): 542-542. |
| 15 | Chan, W. C. and L. C. W. Lam (2013). | Dementia - An 'old' disorder with new perspectives. | East Asian Archives of Psychiatry 23(3): 76-77. |
| 16 | Connelly, P. J. (2007). | Investigating and treating dementia in primary care. | Practitioner 251(1693): 27-36. |
| 17 | Duthie, A. and C. Q. Mountjoy (2000). | The management of the Alzheimer's patient by the general practitioner: A UK perspective. | Primary Care Psychiatry 6(2): 49-55. |
| 18 | El-Badri, N. S. (2009). | Toward optimal health: advising aging women about dementia. Interview by Jodi R. Godfrey. | Journal of Women's Health 18(7): 929-933. |
| 19 | Gandy, S. (2011). | Perspective: prevention is better than cure. | Nature 475(7355): S15. |
| 20 | Gauthier, S. (2001). | Alzheimer's disease: current and future therapeutic perspectives. | Progress in Neuro-Psychopharmacology and Biological Psychiatry 25(1): 73-89. |
| 21 | Hakim, A. M. (2014). | Perspective: silent, but preventable, perils. | Nature 510(7506): S12. |
| 22 | Ho, E. Y. and B. K. Woo (2013). | Dementia knowledge and information seeking of Chinese Americans. | Journal of the American Geriatrics Society 61(4): 647-648. |
| 23 | Jankowiak, J. (2002). | Neurology patient page. Depression may be another risk for Alzheimer's dementia: your doctor can help. | Neurology 59(3): E4-5. |
| 24 | Joyner, N. (2007). | Alzheimer's disease (AD)--what nurses should know. | The Prairie rose 76(1): 23-25; quiz 27. |
| 25 | Keady, J. (1999). | Dementia. | Elderly care 11(1): 21-25; quiz 26-27. |
| 26 | Khalsa, D. S. and C. Gustafson (2014). | Dharma Singh Khalsa, MD: The pillars of Alzheimer's prevention. | Advances in mind-body medicine 28(3): 26-32. |
| 27 | Koch, T. and S. Iliffe (2010). | The role of primary care in the recognition of and response to dementia. | Journal of Nutrition, Health and Aging 14(2): 107-109. |
| 28 | Koenig, B. A. and H. L. Silverberg (1999). | Understanding probabilistic risk in predisposition genetic testing for Alzheimer disease. | Genetic Testing 3(1): 55-63. |
| 29 | Kudo, K. and Y. Arai (1998). | Gloomy boom of anti-dementia campaign in Japanese public health. | Lancet 352(9138): 1477-1478. |
| 30 | Laditka, J. N., R. L. Beard, L. L. Bryant, D. Fetterman, R. Hunter, S. Ivey, R. G. Logsdon, J. R. Sharkey and B. Wu (2009). | Promoting cognitive health: a formative research collaboration of the healthy aging research network. | Gerontologist 49 Suppl 1: S12-17. |
| 31 | Lam, N. H. T. and B. K. P. Woo (2018). | Exploring the role of YouTube in delivering dementia education to older Chinese. | Asian Journal of Psychiatry 31: 25-26. |
| 32 | Lee, J. P. and B. K. P. Woo (2015). | Dementia literacy among Chinese Americans: Family history of dementia only marginally improves understanding of the disease. | Journal of Clinical Gerontology and Geriatrics 6(4): 141-142. |
| 33 | Levine, D. A. (2013). | Young-onset dementia: Unanswered questions and unmet needs. | JAMA Internal Medicine 173(17): 1619-1620. |
| 34 | Lin, A. L., D. A. Butterfield and A. Richardson (2016). | mTOR: Alzheimer's disease prevention for APOE4 carriers. | Oncotarget 7(29): 44873-44874. |
| 35 | Lin, F., D. E. Vance, C. E. Gleason and S. M. Heidrich (2012). | Caring for Older Adults with Mild Cognitive Impairment: An Update for Nurses. | Journal of Gerontological Nursing 38(12): 22-35. |
| 36 | Lindeman, M. A. and K. Smith (2010). | Remote primary health care practitioners' views about priorities in Indigenous dementia research. | Rural and remote health 10(3): 1538. |
| 37 | Machanic, B. I. (2009). | High-dose B vitamin supplements and alzheimer disease. | JAMA 301(10): 1021. |
| 38 | Mack, W. J. (2013). | Looking into the crystal ball for Alzheimer's disease. | Science Translational Medicine 5 (207) (no pagination)(207ec169). |
| 39 | Marchionni, M., S. Caramel and S. Stagnaro (2013). | Inherited real risk of Alzheimer's disease: Bedside diagnosis and primary prevention. | Frontiers in Aging Neuroscience 5 (MAR) (no pagination)(article 13). |
| 40 | McCallum, J. (2015). | Survey identifies key dementia research priorities. | Medical Journal of Australia 202(6): 286-286. |
| 41 | McCartney, M. (2016). | Margaret McCartney: Looking for dementia-in the name of "awareness" | BMJ (Online) 352 (no pagination)(i1621). |
| 42 | Moga, D. C., E. L. Abner and E. S. Brouwer (2015). | Dementia and "obesity paradox": is this for real or are we missing something? An epidemiologist's perspective. | Journal of the American Medical Directors Association 16(1): 78-79. |
| 43 | Musicco, M. and K. Palmer (2013). | News from epidemiological studies on Alzheimer's and Parkinson's disease: A personal perspective. | Journal of Neurology 260(8): 2191-2192. |
| 44 | Padala, K. P., P. R. Padala and W. J. Burke (2011). | Wii-Fit as an adjunct for mild cognitive impairment: Clinical perspectives. | Journal of the American Geriatrics Society 59(5): 932-933. |
| 45 | Perlmutter, D. (2016). | Preventing Alzheimer's Disease. | Journal of the American College of Nutrition 35(8): 732-733. |
| 46 | Piemontese, L. (2017). | New approaches for prevention and treatment of Alzheimer's disease: A fascinating challenge. | Neural Regeneration Research 12(3): 405-406. |
| 47 | Pond, C. D. (2017). | The role of primary care in identification and ongoing management of dementia: A time of transition. | International Psychogeriatrics 29(9): 1409-1411. |
| 48 | Post, S. G. (1995). | Dementia in our midst: the moral community. | Cambridge quarterly of healthcare ethics : CQ : the international journal of healthcare ethics committees 4(2): 142-147. |
| 49 | Powell, G. (2007). | Viewpoint: A new view of Alzheimer's disease. | Drug Topics 151(2). |
| 50 | Prodan, C. I., M. Monnot, R. A. Brumback and E. D. Ross (2007). | Initiating referral in mild cognitive impairment: Who rings the bell? | Journal of the American Geriatrics Society 55(7): 1147-1149. |
| 51 | Ralat, J. (2006). | Increased Awareness of Dementia in Late Life May Improve Quality of Life in Elderly. | CNS Spectrums 11(12): 909. |
| 52 | Roach, P. and J. Keady (2008). | Younger people with dementia: time for fair play. | British Journal of Nursing 17(11): 690. |
| 53 | Robb-Nicholson, C. (2009). | By the way, doctor. The "Women's Health Initiative" found that hormone therapy wasn't helpful for avoiding dementia; there was some suggestion that it might even cause cognitive problems. Am I at risk for dementia by continuing hormone therapy? | Harvard women's health watch 17(3): 8. |
| 54 | Sakurai, T., H. Taniguchi, S. Nambu, M. Tokuda, T. Makibayashi, K. Yokono and H. Endo (2009). | Education of lifestyle risk factors associated with dementia likely to help primary care physicians to improve consultation for elderly people with dementia. | Journal of the American Geriatrics Society 57(12): 2358-2361. |
| 55 | Sawyer, N. (2000). | The state of affairs in Alzheimer disease. | The Case manager 11(5): 61-68. |
| 56 | Schneider, L. S. (2016). | Reduce vascular risk to prevent dementia? | The Lancet 388(10046): 738-740. |
| 57 | Shumaker, S. A., C. Legault, L. Kuller and C. L. Roumie (2004). | Hormone replacement therapy and the risk of dementia: The woman's health initiative memory study. | Journal of Clinical Outcomes Management 11(8): 500-501. |
| 58 | Shute, N. (2004). | A view of dementia. | U.S news & world report. 136(4): 53. |
| 59 | Sisodia, S. S. (1999). | Alzheimer's disease: Perspectives for the new millennium. | Journal of Clinical Investigation 104(9): 1169-1170. |
| 60 | Soros, P., S. Whitehead, J. D. Spence and V. Hachinski (2013). | Opinion: Antihypertensive treatment can prevent stroke and cognitive decline. | Nature Reviews Neurology 9(3): 174-178. |
| 61 | Staessen, J. A., T. Richart and W. H. Birkenhager (2007). | Less atherosclerosis and lower blood pressure for a meaningful life perspective with more brain. | Hypertension 49(3): 389-400. |
| 62 | Steffens, D. C. (2018). | A geriatrics perspective on dementia prevention and treatment. | American Journal of Psychiatry 175(3): 199-201. |
| 63 | Stephenson, J. (2001). | Racial barriers may hamper diagnosis, care of patients with Alzheimer disease. | JAMA 286(7): 779-780. |
| 64 | Strohle, A. and M. A. Rapp (2016). | Prevention of Cognitive Decline: A Physical Exercise Perspective on Brain Health in the Long Run. | Journal of the American Medical Directors Association 17(5): 461-462. |
| 65 | Sturdy, D. (2009). | The National Dementia Strategy: nurses need to lead change. | Nursing older people 21(1): 12-13. |
| 66 | Thomas, S. P. (2012). | Reducing the risk for Alzheimer's. | Issues in mental health nursing 33(2): 65. |
| 67 | Toniolo, S. (2011). | Neuropsychological interventions in stroke survivors: implications for evidence based psychological practice. | Giornale Italiano di Medicina del Lavoro Ed Ergonomia 33(1 Suppl A): A29-36. |
| 68 | Travers, C. M., M. G. Martin-Khan and D. C. Lie (2009). | Dementia risk reduction in primary care: What Australian initiatives can teach us. | Australian Health Review 33(3): 461-466. |
| 69 | Travers, C., M. Martin-Khan and D. Lie (2009). | Barriers and enablers of health promotion, prevention and early intervention in primary care: Evidence to inform the Australian national dementia strategy. | Australasian Journal on Ageing 28(2): 51-57. |
| 70 | Tripathi, R., K. Kumar, R. Balachandar, P. Marimuthu, M. Varghese and S. Bharath (2016). | Cognitive markers of mild cognitive impairment: An Indian experience. | Annals of Indian Academy of Neurology 19(1): 164. |
| 71 | Vamer, J. M. (2006). | Dementia: thief of hearts. | The Alabama nurse 33(3): 26-28; quiz 28-29. |
| 72 | Viscogliosi, G. and V. Marigliano (2013). | Alzheimer's disease: How far have we progressed? Lessons learned from diabetes mellitus, metabolic syndrome, and inflammation. | Journal of the American Geriatrics Society 61(5): 845-846. |
| 73 | Volland, J., A. Fisher and D. Drexler (2015). | Delirium and Dementia in the Intensive Care Unit: Increasing Awareness for Decreasing Risk, Improving Outcomes, and Family Engagement. | Dimensions of critical care nursing : DCCN 34(5): 259-264. |
| 74 | Watson, K. E. and T. Sallam (2017). | MY APPROACH to the Patient With Memory Loss Who Needs a Statin. | Trends in Cardiovascular Medicine 27(2): 158-159. |
| 75 | Westphal, A., W. Hall and E. Chiu (2011). | Opinion enhancing quality of life in dementia. | Asia-Pacific Psychiatry 3(3): 103-106. |
| 76 | Wheldon, M. (2005). | Untangling the confusion. Alzheimer's management today. | Advance for nurse practitioners 13(5): 47-48, 50, 52. |
| 77 | While, A. (2009). | The challenge of dementia. | British journal of community nursing 14(2): 94. |
| 78 | Wise, J. (2010). | Dancing with dementia. | BMJ 340: c1215. |
| 79 | Wollen, K. A. (2010). | Alzheimer's disease: the pros and cons of pharmaceutical, nutritional, botanical, and stimulatory therapies, with a discussion of treatment strategies from the perspective of patients and practitioners. | Alternative Medicine Review 15(3): 223-244. |

*Reason 3: No relevant primary data (n=129)*

| 1 | Abuzinadah, A. R. and L. Cooke (2017). | Neurology Health Advocacy Curriculum: Needs Assessment, Curricular Content and Underlying Components. | Canadian Journal of Neurological Sciences 44(2): 170-176. |
| --- | --- | --- | --- |
| 2 | Allen, J. and J. R. Oyebode (2009). | Having a father with young onset dementia: the impact on well-being of young people. | Dementia 8(4): 455-480. |
| 3 | Annear, M. J., C. Toye, K.-E. J. Elliott, F. McInerney, C. Eccleston and A. Robinson (2017). | Dementia knowledge assessment scale (DKAS): confirmatory factor analysis and comparative subscale scores among an international cohort. | BMC Geriatrics 17: 1-11. |
| 4 | Askari, N., A. C. Bilbrey, I. Garcia Ruiz, M. B. Humber and D. Gallagher-Thompson (2018). | Dementia Awareness Campaign in the Latino Community: A Novel Community Engagement Pilot Training Program with <italic>Promotoras</italic>. | Clinical Gerontologist 41(3): 200-208. |
| 5 | Austrom, M. G., C. Hartwell, P. S. Moore, M. Boustani, H. C. Hendrie and C. M. Callahan (2006). | A care management model for enhancing physician practice for Alzheimer disease in primary care. | Clinical Gerontologist 29(2): 35-43. |
| 6 | Barca, M. L., K. Thorsen, K. Engedal, P. K. Haugen and A. Johannessen (2014). | Nobody asked me how I felt: experiences of adult children of persons with young-onset dementia. | International Psychogeriatrics 26(12): 1935-1944. |
| 7 | Bethell, J., D. Pringle, L. W. Chambers, C. Cohen, E. Commisso, K. Cowan, P. Fehr, A. Laupacis, P. Szeto and K. S. McGilton (2018). | Patient and Public Involvement in Identifying Dementia Research Priorities. | Journal of the American Geriatrics Society 66(8): 1608-1612. |
| 8 | Boessen, A. B. C. G., J. Vermeulen and L. P. de Witte (2017). | Acceptance and usability of a home-based monitoring tool of health indicators in children of people with dementia: A proof of principle (POP) study. | Patient Preference and Adherence 11: 1317-1324. |
| 9 | Bond, J., N. Graham, A. Padovani, J. Mackell, S. Knox and J. Atkinson (2010). | Screening for cognitive impairment, Alzheimer's disease and other dementias: opinions of European caregivers, payors, physicians and the general public. | Journal of Nutrition, Health and Aging 14(7): 558-562. |
| 10 | Bond, K. S., A. F. Jorm, B. A. Kitchener, C. M. Kelly and K. J. Chalmers (2016). | Development of guidelines for family and non-professional helpers on assisting an older person who is developing cognitive impairment or has dementia: a Delphi expert consensus study. | BMC geriatrics 16: 129. |
| 11 | Brandt, J., C. Sullivan, L. E. Burrell, 2nd, M. Rogerson and A. Anderson (2013). | Internet-based screening for dementia risk. | PLoS ONE 8(2): e57476. |
| 12 | Bush, C., J. Kozak and T. Elmslie (1997). | Screening for cognitive impairment in the elderly. | Canadian family physician Medecin de famille canadien 43: 1763-1768. |
| 13 | Cahill, S., M. Clark, H. O'Connell, B. Lawlor, R. F. Coen and C. Walsh (2008). | The attitudes and practices of general practitioners regarding dementia diagnosis in Ireland. | International Journal of Geriatric Psychiatry 23(7): 663-669. |
| 14 | Canabate, P., G. Martinez, M. Rosende-Roca, M. Moreno, S. Preckler, S. Valero, O. Sotolongo, I. Hernandez, M. Alegret, G. Ortega, A. Espinosa, A. Mauleon, L. Vargas, O. Rodriguez, C. Abdelnour, D. Sanchez, E. Martin, A. Ruiz, L. Tarraga and M. Boada (2017). | Social Representation of Dementia: An Analysis of 5,792 Consecutive Cases Evaluated in a Memory Clinic. | Journal of Alzheimer's Disease 58(4): 1099-1108. |
| 15 | Carter, R. E., D. A. Rose, Y. Y. Palesch and J. E. Mintzer (2004). | Alzheimer's disease in the family practice setting: assessment of a screening tool. | Primary Care Companion to the Journal of Clinical Psychiatry 6(6): 234-238. |
| 16 | Castro-Rojas, M. D. (2018). | Willingness and performance of older adults using Information ana Communication Technologies for cognitive activity and social interaction. | Gerontechnology 17(3): 160-173. |
| 17 | Chodosh, J., D. B. Petitti, M. Elliott, R. D. Hays, V. C. Crooks, D. B. Reuben, J. G. Buckwalter and N. Wenger (2004). | Physician recognition of cognitive impairment: evaluating the need for improvement. | Journal of the American Geriatrics Society 52(7): 1051-1059. |
| 18 | Chow, S., R. Chow, C. Yu, O. Nadalini, D. Krcmar, C. DeAngelis and N. Herrmann (2018). | Dementia awareness for high school students: A pilot program. | International Public Health Journal 10(2): 189-195. |
| 19 | Clarke, C. L., J. Keady, H. Wilkinson, C. E. Gibb, A. Luce, A. Cook and L. Williams (2010). | Dementia and risk: contested territories of everyday life. | Journal of Nursing and Healthcare of Chronic Illnesses 2(2): 102-112. |
| 20 | Cooper, C., P. Bebbington, J. Lindesay, H. Meltzer, S. McManus, R. Jenkins and G. Livingston (2011). | The meaning of reporting forgetfulness: a cross-sectional study of adults in the English 2007 Adult Psychiatric Morbidity Survey. | Age and Ageing 40(6): 711-717. |
| 21 | Corwin, S. J., J. N. Laditka, S. B. Laditka, S. Wilcox, R. Liu, S. J. Corwin, J. N. Laditka, S. B. Laditka, S. Wilcox and R. Liu (2009). | Attitudes on aging well among older African Americans and whites in South Carolina. | Preventing Chronic Disease 6(4): A113-A113. |
| 22 | Dal Bello-Haas, V. P. M., M. E. O’Connell, D. G. Morgan and M. Crossley (2014). | Lessons learned: feasibility and acceptability of a telehealth-delivered exercise intervention for rural- dwelling individuals with dementia and their caregivers. | Rural and Remote Health 14(3): 1-11. |
| 23 | Danner, D. D., C. D. Smith, P. Jessa and J. Hudson (2008). | African Americans with Memory Loss: Findings from a Community Clinic in Lexington, Kentucky. | Nursing Clinics of North America 43(3): 437-447. |
| 24 | Daviglus, M. L., C. C. Bell, W. Berrettini, P. E. Bowen, E. S. Connolly Jr, N. J. Cox, J. M. Dunbar-Jacob, E. C. Granieri, G. Hunt, K. McGarry, D. Patel, A. L. Potosky, E. Sanders-Bush, D. Silberberg and M. Trevisan (2010). | NIH state-of-the-science conference statement: Preventing Alzheimer's disease and cognitive decline. | NIH consensus and state-of-the-science statements 27(4): 1-30. |
| 25 | Davis, D. (2015). | Ethical Issues in Interpretation of Risk, from the Perspective of a Research Subject. | Narrative Inquiry in Bioethics 5(3): 203-206. |
| 26 | de Haro, A. E., S. M. de Miguel Lopez and J. R. Sanchez (2016). | Mild cognitive impairment in elderly users of municipal centers of the Region of Murcia (Spain). | Anales de Psicologia 32(1): 234-240. |
| 27 | Dhikav, V., P. Singh and K. S. Anand (2013). | Medication adherence survey of drugs useful in prevention of dementia of Alzheimer's type among Indian patients. | International Psychogeriatrics 25(9): 1409-1413. |
| 28 | Di Bona, L., J. Wenborn, B. Field, S. M. Hynes, R. Ledgerd, G. Mountain and T. Swinson (2017). | Enablers and challenges to occupational therapists' research engagement: A qualitative study. | British Journal of Occupational Therapy 80(11): 642-650. |
| 29 | Duane, F. M., D. P. Goeman, C. J. Beanland and S. H. Koch (2015). | The role of a clinical nurse consultant dementia specialist: A qualitative evaluation. | Dementia 14(4): 436-449. |
| 30 | Ellison, J. M. (2008). | A 60-year-old woman with mild memory impairment: review of mild cognitive impairment. | JAMA 300(13): 1566-1574. |
| 31 | Forette, F., A. Padovani, K. Berthet and S. Knox (2010). | Implications of the Impact Survey for payors across Europe. | Journal of Nutrition, Health and Aging 14(7): 553-557. |
| 32 | Fowler, N. R., A. J. Perkins, H. A. Turchan, A. Frame, P. Monahan, S. Gao and M. A. Boustani (2015). | Older primary care patients' attitudes and willingness to screen for dementia. | Journal of Aging Research 2015 (no pagination)(423265). |
| 33 | Fox, K. R., A. Stathi, J. McKenna and M. G. Davis (2007). | Physical activity and mental well-being in older people participating in the Better Ageing Project. | European Journal of Applied Physiology 100(5): 591-602. |
| 34 | Frost, S., L. B. Myers and S. P. Newman (2001). | Genetic screening for Alzheimer's disease: what factors predict intentions to take a test? | Behavioral Medicine 27(3): 101-109. |
| 35 | Gallucci, M., S. Mazzuco, F. Ongaro, E. Giorgi, P. Mecocci, M. Cesari, D. Albani, G. Forloni, E. Durante, G. Gajo, A. Zanardo, M. Siculi, L. Caberlotto and C. Regini (2013). | Body mass index, lifestyles, physical performance and cognitive decline: The 'Treviso Longeva (Trelong)' study. | Journal of Nutrition, Health and Aging 17(4): 378-384. |
| 36 | Galvin, J. E., Q. Fu, J. T. Nguyen, C. Glasheen and D. P. Scharff (2008). | Psychosocial determinants of intention to screen for Alzheimer's disease. | Alzheimer's and Dementia 4(5): 353-360. |
| 37 | Gildengers, A. G., M. A. Butters, S. M. Albert, S. J. Anderson, M. A. Dew, K. Erickson, L. Garand, J. F. Karp, M. H. Lockovich, J. Morse and C. F. Reynolds, III (2016). | Design and implementation of an intervention development study: Retaining Cognition while Avoiding Late-Life Depression (ReCALL). | American Journal of Geriatric Psychiatry 24(6): 444-454. |
| 38 | Gooding, H. C., E. L. Linnenbringer, J. Burack, J. S. Roberts, R. C. Green and B. B. Biesecker (2006). | Genetic susceptibility testing for Alzheimer disease: motivation to obtain information and control as precursors to coping with increased risk. | Patient Education and Counseling 64(1-3): 259-267. |
| 39 | Grill, J. D., R. J. Bateman, V. Buckles, A. Oliver, J. C. Morris, C. L. Masters, W. E. Klunk and J. M. Ringman (2015). | A survey of attitudes toward clinical trials and genetic disclosure in autosomal dominant Alzheimer's disease. | Alzheimer's Research and Therapy 7 (1) (no pagination)(50). |
| 40 | Guan, Y., D. L. Roter, J. L. Wolff, L. N. Gitlin, K. D. Christensen, J. S. Roberts, R. C. Green and L. H. Erby (2017). | The impact of genetic counselors' use of facilitative strategies on cognitive and emotional processing of genetic risk disclosure for Alzheimer's disease. | Patient education and counseling, 101(5), 817-823. |
| 41 | Haapala, I., A. Carr and S. Biggs (2018). | Differences in priority by age group and perspective: Implications for public health education and campaigning in relation to dementia. | International Psychogeriatrics 30(11): 1583-1591. |
| 42 | Hansen, K. T., C. McDonald, S. O'Hara, L. Post, S. Silcox and I. A. Gutmanis (2017). | A formative evaluation of a nurse practitioner-led interprofessional geriatric outpatient clinic. | Journal of Interprofessional Care 31(4): 546-549. |
| 43 | Harada, K., S. Lee, H. Shimada, S. Lee, S. Bae, Y. Anan, K. Harada and T. Suzuki (2017). | Psychological predictors of participation in screening for cognitive impairment among community-dwelling older adults. | Geriatrics and Gerontology International 17(8): 1197-1204. |
| 44 | Hietaranta-Luoma, H. L., H. T. Luomala, H. Puolijoki and A. Hopia (2015). | Using ApoE Genotyping to Promote Healthy Lifestyles in Finland - Psychological Impacts: Randomized Controlled Trial.[Erratum appears in J Genet Couns. 2016 Dec;25(6):1345-1346; PMID: 27680565]. | Journal of Genetic Counseling 24(6): 908-921. |
| 45 | Ho, Y.-C. and A. S. Chan (2005). | Comparing the Effects of Mahjong Playing and Reading on Cognitive Reserve of the Elderly. | Journal of Psychology in Chinese Societies 6(1): 5-26. |
| 46 | Hsu, M. C., W. Moyle, D. Creedy and L. Venturato (2005). | An investigation of aged care mental health knowledge of Queensland aged care nurses. | International Journal of Mental Health Nursing 14(1): 16-23. |
| 47 | Hunsaker, A., C. E. Sarles, D. Rosen, J. H. Lingler, M. B. Johnson, L. Morrow and J. Saxton (2011). | Exploring the reasons urban and rural-dwelling older adults participate in memory research. | American Journal of Alzheimer's Disease and Other Dementias 26(3): 227-234. |
| 48 | Hutchinson, K., C. Roberts, M. Daly, C. Bulsara and S. Kurrle (2016). | Empowerment of young people who have a parent living with dementia: a social model perspective. | International Psychogeriatrics 28(4): 657-668. |
| 49 | Hutchinson, K., C. Roberts, S. Kurrle and M. Daly (2016). | The emotional well-being of young people having a parent with younger onset dementia. | Dementia 15(4): 609-628. |
| 50 | Iliffe, S. and J. Manthorpe (2004). | The recognition of and response to dementia in the community: lessons for professional development. | Learning in Health and Social Care 3(1): 5-16. |
| 51 | Iliffe, S., J. Manthorpe and A. Eden (2003). | Sooner or later? Issues in the early diagnosis of dementia in general practice: A qualitative study. | Family Practice 20(4): 376-381. |
| 52 | Isaacson, R. S., A. Seifan, C. L. Haddox, M. Mureb, A. Rahman, O. Scheyer, K. Hackett, E. Caesar, J. L. Chen, J. Isaacson, M. McInnis, L. Mosconi and J. Safdieh (2018). | Using social media to disseminate education about Alzheimer's prevention & treatment: a pilot study on Alzheimer's Universe (www.AlzU.org). | Journal of Communication in Healthcare 11(2): 106-113. |
| 53 | Jenkins, A., A. Tales, J. Tree and A. Bayer (2015). | Are We Ready? The Construct of Subjective Cognitive Impairment and its Utilization in Clinical Practice: A Preliminary UK-Based Service Evaluation. | Journal of Alzheimer's Disease 48 Suppl 1: S25-31. |
| 54 | Johannessen, A., K. Engedal and K. Thorsen (2016). | Coping efforts and resilience among adult children who grew up with a parent with young-onset dementia: a qualitative follow-up study. | International journal of qualitative studies on health and well-being 11: 30535. |
| 55 | Jones, R. W., J. Mackell, K. Berthet and S. Knox (2010). | Assessing attitudes and behaviours surrounding Alzheimer's disease in Europe: key findings of the Important Perspectives on Alzheimer's Care and Treatment (IMPACT) survey. | Journal of Nutrition, Health and Aging 14(7): 525-530. |
| 56 | Jorm, A. F., P. Butterworth, K. J. Anstey, H. Christensen, S. Easteal, J. Maller, K. A. Mather, R. I. Turakulov, W. Wen and P. Sachdev (2004). | Memory complaints in a community sample aged 60-64 years: associations with cognitive functioning, psychiatric symptoms, medical conditions, APOE genotype, hippocampus and amygdala volumes, and white-matter hyperintensities. | Psychological Medicine 34(8): 1495-1506. |
| 57 | Justiss, M. D., M. Boustani, C. Fox, C. Katona, A. J. Perkins, P. J. Healey, G. Sachs, S. Hui, C. M. Callahan, H. C. Hendrie, E. Scott, M. D. Justiss, M. Boustani, C. Fox, C. Katona, A. J. Perkins, P. J. Healey, G. Sachs, S. Hui and C. M. Callahan (2009). | Patients' attitudes of dementia screening across the Atlantic. | International Journal of Geriatric Psychiatry 24(6): 632-637. |
| 58 | Kally, Z., D. L. Cherry, S. Howland and M. Villarruel (2014). | Asian Pacific Islander Dementia Care Network: A model of care for underserved communities. | Journal of Gerontological Social Work 57(6-7): 710-727. |
| 59 | LaBond, V., K. R. Barber and I. J. Golden (2014). | Sports-related head injuries in students: parents' knowledge, attitudes, and perceptions. | NASN school nurse 29(4): 194-199. |
| 60 | Laditka, J. N., S. B. Laditka, R. Liu, A. E. Price, B. Wu, D. B. Friedman, S. J. Corwin, J. R. Sharkey, W. Tseng, R. Hunter and R. G. Logsdon (2011). | Older adults' concerns about cognitive health: commonalities and differences among six United States ethnic groups. | Ageing and Society 31(7): 1202-1228. |
| 61 | Laforce Jr, R. and S. McLean (2005). | Knowledge and fear of developing alzheimer's disease in a sample of healthy adults. | Psychological Reports 96(1): 204-206. |
| 62 | Laforest, S., A. Lorthios-Guilledroit, K. Nour, M. Parisien, M. Fournier, D. Ellemberg, D. Guay, C. E. Desgagnes-Cyr and N. Bier (2017). | Attitudes and lifestyle changes following Jog your Mind: results from a multi-factorial community-based program promoting cognitive vitality among seniors. | Health Education Research 32(2): 184-196. |
| 63 | Langdon, R., M. Johnson, V. Carroll and G. Antonio (2013). | Assessment of the elderly: it's worth covering the risks. | Journal of Nursing Management 21(1): 94-105. |
| 64 | Liebel, D. V., B. A. Powers, B. Friedman and N. M. Watson (2012). | Barriers and facilitators to optimize function and prevent disability worsening: a content analysis of a nurse home visit intervention. | Journal of Advanced Nursing 68(1): 80-93. |
| 65 | Lineweaver, T. T., M. W. Bondi, D. Galasko and D. P. Salmon (2014). | Effect of knowledge of APOE genotype on subjective and objective memory performance in healthy older adults. | American Journal of Psychiatry 171(2): 201-208. |
| 66 | Lingler, J. H., M. A. Butters, A. L. Gentry, H. Lu, A. E. Hunsaker, W. E. Klunk, M. K. Mattos, L. A. Parker, J. S. Roberts, R. Schulzb, L. Hu and R. Schulz (2016). | Development of a Standardized Approach to Disclosing Amyloid Imaging Research Results in Mild Cognitive Impairment. | Journal of Alzheimer's Disease 52(1): 17-24. |
| 67 | Liu, D., L. Hinton, C. Tran, D. Hinton, J. C. Barker, D. Liu, L. Hinton, C. Tran, D. Hinton and J. C. Barker (2008). | Reexamining the relationships among dementia, stigma, and aging in immigrant Chinese and Vietnamese family caregivers. | Journal of Cross-Cultural Gerontology 23(3): 283-299. |
| 68 | Logsdon, R. G., S. M. McCurry, K. C. Pike and L. Teri (2009). | Making physical activity accessible to older adults with memory loss: a feasibility study. | Gerontologist 49 Suppl 1: S94-99. |
| 69 | Lu, Y. Y.-F. and J. E. Haase (2011). | Content validity and acceptability of the daily enhancement of meaningful activity program: intervention for mild cognitive impairment patient-spouse dyads. | Journal of Neuroscience Nursing 43(6): 317-328. |
| 70 | Lysack, C., P. Lichtenberg and B. Schneider (2011). | Effect of a DVD intervention on therapists' mental health practices with older adults. | American Journal of Occupational Therapy 65(3): 297-305. |
| 71 | Maeck, L., S. Haak, A. Knoblauch and G. Stoppe (2007). | Early diagnosis of dementia in primary care: A representative eight-year follow-up study in Lower Saxony, Germany. | International Journal of Geriatric Psychiatry 22(1): 23-31. |
| 72 | Mak, W. and S. Sorensen (2012). | Trajectories of preparation for future care among first-degree relatives of Alzheimer's disease patients: an ancillary study of ADAPT. | The Gerontologist 52(4): 531-540. |
| 73 | Manthorpe, J., K. Samsi, S. Campbell, C. Abley, J. Keady, J. Bond, S. Watts, L. Robinson, J. Warner, S. Iliffe, J. Manthorpe, K. Samsi, S. Campbell, C. Abley, J. Keady, J. Bond, S. Watts, L. Robinson, J. Warner and S. Iliffe (2013). | From forgetfulness to dementia: clinical and commissioning implications of diagnostic experiences. | British Journal of General Practice 63(606): 69-75. |
| 74 | McDonald, A. (2010). | The impact of the 2005 Mental Capacity Act on social workers' decision making and approaches to the assessment of risk. | British Journal of Social Work 40(4): 1229-1246. |
| 75 | Milne, A. J., K. Hamilton-West and E. Hatzidimitriadou (2005). | GP attitudes to early diagnosis of dementia: evidence of improvement. | Aging and Mental Health 9(5): 449-455. |
| 76 | Milne, R., E. Bunnik, A. Diaz, E. Richard, S. Badger, D. Gove, J. Georges, K. Fauria, J. L. Molinuevo, K. Wells, C. Ritchie and C. Brayne (2018). | Perspectives on Communicating Biomarker-Based Assessments of Alzheimer's Disease to Cognitively Healthy Individuals. | Journal of Alzheimer's Disease 62(2): 487-498. |
| 77 | Mitchell, S., S. H. Ridley, R. M. Sancho and M. Norton (2017). | The future of dementia risk reduction research: barriers and solutions. | Journal of Public Health 39(4): e275-e281. |
| 78 | Molinuevo, J. L., L. Pintor, J. M. Peri, A. Lleó, R. Oliva, T. Marcos and R. Blesa (2005). | Emotional reactions to predictive testing in Alzheimer's disease and other inherited dementias. | American Journal of Alzheimer's Disease and Other Dementias 20(4): 233-238. |
| 79 | Murray, M. D., K. A. Lane, S. Gao, R. M. Evans, F. W. Unverzagt, K. S. Hall and H. Hendrie (2002). | Preservation of cognitive function with antihypertensive medications: a longitudinal analysis of a community-based sample of African Americans. | Archives of Internal Medicine 162(18): 2090-2096. |
| 80 | Neilson, J. (1999). | A patient's perspective on genetic counseling and predictive testing for Alzheimer's disease. | Journal of Genetic Counseling 8(1): 37-46. |
| 81 | Neumann, P. J., J. T. Cohen, J. K. Hammitt, T. W. Concannon, H. R. Auerbach, C. Fang and D. M. Kent (2012). | Willingness-to-pay for predictive tests with no immediate treatment implications: A survey of US residents. | Health Economics 21(3): 238-251. |
| 82 | Ott, B. R., M. A. Pelosi, G. Tremont and P. J. Snyder (2016). | A survey of knowledge and views concerning genetic and amyloid positron emission tomography status. | Alzheimer's and Dementia: Translational Research and Clinical Interventions 2(1): 23-29. |
| 83 | Palacios-Cena, D., C. Alvarez-Lopez, C. Cachon-Perez and C. Alonso-Blanco (2009). | Early detection of functional and cognitive decline after hospital discharge: The role of community nursing and multidisciplinary teams. | Journal of Gerontological Nursing 35(9): 13-18. |
| 84 | Palmer, A. C. and B. M. Withee (1996). | Dementia care: effects of behavioral intervention training on staff perceptions of their work in veterans' nursing home. | Geriatric nursing 17(3): 137-140. |
| 85 | Park, D. C. and S. J. Birge (2002). | Toward optimal health: the experts discuss cognitive function and memory changes. | Journal of Women's Health 11(8): 683-689. |
| 86 | Pentzek, M., H. H. Abholz, M. Ostapczuk, A. Altiner, A. Wollny, A. Fuchs, M. Pentzek, H.-H. Abholz, M. Ostapczuk, A. Altiner, A. Wollny and A. Fuchs (2009). | Dementia knowledge among general practitioners: first results and psychometric properties of a new instrument. | International Psychogeriatrics 21(6): 1105-1115. |
| 87 | Pesonen, H. M., A. M. Remes and A. Isola (2013). | Diagnosis of dementia as a turning point among Finnish families: a qualitative study. | Nursing and health sciences 15(4): 489-496. |
| 88 | Phillipson, L., C. A. Magee, S. C. Jones and E. Skladzien (2014). | Correlates of dementia attitudes in a sample of middle-aged Australian adults. | Australasian Journal on Ageing 33(3): 158-163. |
| 89 | Pitre, N., S. Stewart, S. Adams, T. Bedard and S. Landry (2007). | The use of puppets with elementary school children in reducing stigmatizing attitudes towards mental illness. | Journal of Mental Health 16(3): 415-429. |
| 90 | Post, S. G., P. J. Whitehouse, R. H. Binstock, T. D. Bird, S. K. Eckert, L. A. Farrer, L. M. Fleck, A. D. Gaines, E. T. Juengst, H. Karlinsky, S. Miles, T. H. Murray, K. A. Quaid, N. R. Relkin, A. D. Roses, P. H. St George-Hyslop, G. A. Sachs, B. Steinbock, E. F. Truschke and A. B. Zinn (1997). | The clinical introduction of genetic testing for Alzheimer disease. An ethical perspective. | JAMA 277(10): 832-836. |
| 91 | Prasad, K., H. Gupta, S. Bharath, O. Prakash, P. T. Sivakumar, C. N. Kumar and M. Varghese (2009). | Clinical practice with antidementia and antipsychotic drugs: audit from a geriatric clinic in India. | Indian Journal of Psychiatry 51(4): 272-275. |
| 92 | Qualls, S. H., K. J. Klebe, K. Berryman, A. Williams, L. Phillips, H. Layton, K. Hiroto, M. Stephens, L. Anderson and M. Rogers (2015). | Motivational and cognitive pathways to medical help-seeking for Alzheimer's disease: a cognitive impairment response model. | Journals of Gerontology Series B-Psychological Sciences and Social Sciences 70(1): 57-66. |
| 93 | Rickles, N. M., J. B. Skelton, J. Davis and J. Hopson (2014). | Cognitive memory screening and referral program in community pharmacies in the United States. | International Journal of Clinical Pharmacy 36(2): 360-367. |
| 94 | Roberts, J. S., M. Barber, T. M. Brown, L. A. Cupples, L. A. Farrer, S. A. LaRusse, S. G. Post, K. A. Quaid, L. D. Ravdin, N. R. Relkin, A. D. Sadovnick, P. J. Whitehouse, J. L. Woodard and R. C. Green (2004). | Who seeks genetic susceptibility testing for Alzheimer's disease? Findings from a multisite, randomized clinical trial. | Genetics in Medicine 6(4): 197-203. |
| 95 | Roberts, J. S., S. A. LaRusse, H. Katzen, P. J. Whitehouse, M. Barber, S. G. Post, N. Relkin, K. Quaid, R. H. Pietrzak, L. A. Cupples, L. A. Farrer, T. Brown and R. C. Green (2003). | Reasons for seeking genetic susceptibility testing among first-degree relatives of people with Alzheimer disease. | Alzheimer Disease and Associated Disorders 17(2): 86-93. |
| 96 | Robillard, J. M., D. Roskams-Edris, B. Kuzeljevic and J. Illes (2014). | Prevailing public perceptions of the ethics of gene therapy. | Human Gene Therapy 25(8): 740-746. |
| 97 | Robillard, J. M., T. W. Johnson, C. Hennessey, B. L. Beattie and J. Illes (2013). | Aging 2.0: health information about dementia on Twitter. | PLoS ONE 8(7): e69861. |
| 98 | Robinson, P., S. Ekman, A. I. Meleis, B. Winblad and L. Wahlund (1997). | Suffering in silence: the experience of early memory loss. | Health Care in Later Life 2(2): 107-120. |
| 99 | Rönngren, Y. M., A. Björk, D. Haage and L. Kristiansen (2014). | LIFEHOPE. EU: lifestyle and healthy outcome in physical education. | Journal of Psychiatric and Mental Health Nursing 21(10): 924-930. |
| 100 | Roth, C. P., D. A. Ganz, L. Nickels, D. Martin, R. Beckman and N. S. Wenger (2012). | Nurse Care Manager Contribution to Quality of Care in a Dual-Eligible Special Needs Plan. | Journal of Gerontological Nursing 38(7): 44-54. |
| 101 | Sano, M., S. Egelko, C. W. Zhu, C. Li, M. C. Donohue, S. Ferris, J. Kaye, J. C. Mundt, C. K. Sun, P. S. Aisen and H. H. Feldman (2018). | Participant satisfaction with dementia prevention research: Results from Home-Based Assessment trial. | Alzheimer's and Dementia 14(11): 1397-1405. |
| 102 | Schelp, A. O., A. B. Nieri, P. T. H. Filho, A. M. Bales and C. L. Mendes-Chiloff (2008). | Public awareness of dementia: A study in Botucatu, a medium-sized city in the State of Sao Paulo, Brazil. | Dementia and Neuropsychologia 2(3): 192-196. |
| 103 | Scherer, Y. K., S. A. Bruce, C. A. Montgomery and L. S. Ball (2008). | A challenge in academia: meeting the healthcare needs of the growing number of older adults. | Journal of the American Academy of Nurse Practitioners 20(9): 471-476. |
| 104 | Setia, M., A. M. Islam, J. P. Thompson and D. B. Matchar (2011). | Stakeholders' perspective on issues and challenges associated with care and treatment of aging-related cognitive impairment disorders in Singapore. | International Psychogeriatrics 23(9): 1421-1432. |
| 105 | Shinan-Altman, S. and P. Werner (2017). | Is there an association between help-seeking for early detection of Alzheimer's disease and illness representations of this disease among the lay public? | International Journal of Geriatric Psychiatry 32(12): e100-e106. |
| 106 | States, R. A., W. M. Susman, L. F. Riquelme, E. M. Godwin and E. Greer (2006). | Community health education: reaching ethnically diverse elders. | Journal of Allied Health 35(4): 215-222. |
| 107 | Steinbart, E. J., C. O. Smith, P. Poorkaj and T. D. Bird (2001). | Impact of DNA testing for early-onset familial Alzheimer disease and frontotemporal dementia. | Archives of Neurology 58(11): 1828-1831. |
| 108 | Tannenbaum, C. and N. Mayo (2003). | Women's health priorities and perceptions of care: a survey to identify opportunities for improving preventative health care delivery for older women. | Age and Ageing 32(6): 626-635. |
| 109 | Teel, C. S. (2004). | Rural practitioners' experiences in dementia diagnosis and treatment. | Aging and Mental Health 8(5): 422-429. |
| 110 | Tibben, A., M. Stevens, G. M. de Wert, M. F. Niermeijer, C. M. van Duijn and J. C. van Swieten (1997). | Preparing for presymptomatic DNA testing for early onset Alzheimer's disease/cerebral haemorrhage and hereditary Pick disease. | Journal of Medical Genetics 34(1): 63-72. |
| 111 | Tu, Q., B. Ding, X. Yang, S. Bai, J. Tu, X. Liu, R. Wang, J. Tao, H. Jin, Y. Wang and X. Tang (2014). | The current situation on vascular cognitive impairment after ischemic stroke in Changsha. | Archives of Gerontology and Geriatrics 58(2): 236-247. |
| 112 | Van Hout, H., M. Vernooij-Dassen, K. Bakker, M. Blom and R. Grol (2000). | General practitioners on dementia: Tasks, practices and obstacles. | Patient Education and Counseling 39(2-3): 219-225. |
| 113 | van Vliet, D., M. E. de Vugt, C. Bakker, R. T. C. M. Koopmans, Y. A. L. Pijnenburg, M. J. F. J. Vernooij-Dassen and F. R. J. Verhey (2011). | Caregivers' perspectives on the pre-diagnostic period in early onset dementia: a long and winding road. | International Psychogeriatrics 23(9): 1393-1404. |
| 114 | Vaskinn, A., I. Wilsgård, A. Holm, R. Wootton and B. Elvevåg (2013). | A feasibility study of a telephone-based screening service for mild cognitive impairment and its uptake by elderly people. | Journal of Telemedicine and Telecare 19(1): 5-10. |
| 115 | Vernooij-Dassen, M. J. F. J., E. D. Moniz-Cook, R. T. Woods, J. De Lepeleire, A. Leuschner, O. Zanetti, J. de Rotrou, G. Kenny, M. Franco, V. Peters and S. Iliffe (2005). | Factors affecting timely recognition and diagnosis of dementia across Europe: From awareness to stigma. | International Journal of Geriatric Psychiatry 20(4): 377-386. |
| 116 | Washburn, A. M., J. Luxenberg, M. Brod, M. Steinhauer and M. Katsap (2001). | A club of friends: enrolling nursing home residents in an adult day program. | Journal of the American Medical Directors Association 2(5): 225-229. |
| 117 | Welkenhuysen, M. and G. Evers-Kiebooms (2002). | General practitioners and predictive genetic testing for late-onset diseases in Flanders: what are their opinions and do they want to be involved? | Community Genetics 5(2): 128-137. |
| 118 | Wenger, N. S., C. P. Roth, P. G. Shekelle, R. T. Young, D. H. Solomon, C. J. Kamberg, J. T. Chang, R. Louie, T. Higashi, C. H. MacLean, J. Adams, L. C. Min, K. Ransohoff, M. Hoffing and D. B. Reuben (2009). | A practice-based intervention to improve primary care for falls, urinary incontinence, and dementia. | Journal of the American Geriatrics Society 57(3): 547-555. |
| 119 | Werner, P. (2003). | Knowledge about symptoms of Alzheimer's disease: correlates and relationship to help-seeking behavior. | International Journal of Geriatric Psychiatry 18(11): 1029-1036. |
| 120 | Werner, P. (2005). | Social distance towards a person with Alzheimer's disease. | International Journal of Geriatric Psychiatry 20(2): 182-188. |
| 121 | Werner, P. and M. Davidson (2004). | Emotional reactions of lay persons to someone with Alzheimer's disease. | International Journal of Geriatric Psychiatry 19(4): 391-397. |
| 122 | Wilkinson, H., D. Kerr and C. Rae (2003). | People with a learning disability: their concerns about dementia. | Journal of Dementia Care 11(1): 27-29. |
| 123 | Williams, M. M., D. P. Scharff, K. J. Mathews, J. S. Hoffsuemmer, P. Jackson, J. C. Morris, D. F. Edwards, M. M. Williams, D. P. Scharff, K. J. Mathews, J. S. Hoffsuemmer, P. Jackson, J. C. Morris and D. F. Edwards (2010). | Barriers and facilitators of African American participation in Alzheimer disease biomarker research. | Alzheimer Disease and Associated Disorders 24: S24-29. |
| 124 | Williamson, J. and S. LaRusse (2004). | Genetics and genetic counseling: recommendations for Alzheimer's disease, frontotemporal dementia, and Creutzfeldt-Jakob disease. | Current Neurology and Neuroscience Reports 4(5): 351-357. |
| 125 | Wuthrich, V. M., R. M. Rapee, B. Draper, H. Brodaty, L. F. Low and S. L. Naismith (2018). | Reducing risk factors for cognitive decline through psychological interventions: A pilot randomized controlled trial. | International Psychogeriatrics, 1-11. |
| 126 | Yan, E., A. Wong, W. M. Chan and T. Kwok (2006). | Misconceptions about dementia and its implication for willingness to seek treatment and knowledge in dementia related information. | Hong Kong Practitioner 28(3): 108-113. |
| 127 | Zheng, X. and B. K. P. Woo (2017). | E-mental health in ethnic minority: A comparison of youtube and talk-based educational workshops in dementia. | Asian Journal of Psychiatry 25: 246-248. |
| 128 | Zick, C. D., C. J. Mathews, J. S. Roberts, R. Cook-Deegan, R. J. Pokorski and R. C. Green (2005). | Genetic testing for Alzheimer's disease and its impact on insurance purchasing behavior. | Health Affairs 24(2): 483-490. |
| 129 | Zick, C. D., K. R. Smith and R. N. Mayer (2016). | Planning Ahead or Living a Day at a Time? A Family History of AD and Retirement Planning. | American Journal of Alzheimer's Disease and Other Dementias 31(6): 516-523. |

Part B: Supplemental search (n=11 full-text articles excluded)

*Reason 1: Thesis (n=1)*

| 1 | Yemm, H. (2017). | Cognitive impairment in later life: Understanding lay, professional and memory service user perspectives | Doctoral dissertation, University of Worcester |
| --- | --- | --- | --- |

*Reason 2: No primary data (n=1)*

| 1 | Tang, E. Y. H. and Robinson, A. L. (2014). | Dementia and primary care: views of future general practitioners | Education for Primary Care, 25, 360– 361 |
| --- | --- | --- | --- |

*Reason 3: No relevant primary data (n=9)*

| 1 | Ahmad, S., Orrell, M., Iliffe, S. and Gracie, A. (2010). | GPs’ attitudes, awareness, and practice regarding early diagnosis of dementia | British Journal of General Practice, 60, e360–e365 |
| --- | --- | --- | --- |
| 2 | Anderson, L. A. and R. Egge (2014). | Expanding efforts to address Alzheimer's disease: the Healthy Brain Initiative. | Alzheimer's & Dementia, 10, no. 5, S453-S456 |
| 3 | Cahill, S. C. M., Walsh, C., O’Connell, H. and Lawlor, B. (2006). | Dementia in primary care: the first survey of Irish general practitioners | International Journal of Geriatric Psychiatry, 21, 319–324 |
| 4 | Jennings, A. A., S. Boyle and T. Foley (2018). | The development and evaluation of an online dementia resource for primary care based health professionals. | Internet Interventions, 11, 47-52 |
| 5 | Larson, J. S., et al. (2004). | An Evaluation of Provider Educational Needs in Geriatric Care. | Evaluation & the Health Professions 27(1): 95-103. |
| 6 | Mason, R., K. Doherty and C. Eccleston (2019). | General practitioners attitude and confidence scale for dementia (GPACS-D): confirmatory factor analysis and comparative subscale scores among GPs and supervisors. | BMC family practice, 20(1): 6. |
| 7 | Robinson, B. E., et al. (2001). | Physician confidence and interest in learning more about common geriatric topics: a needs assessment. | Journal of the American Geriatrics Society 49(7): 963-967. |
| 8 | Turner, S. et al. (2004). | General practitioners’ knowledge, confidence and attitudes in the diagnosis and management of dementia | Age and Ageing, 33, 461– 467 |
| 9 | Werner, P. (2014). | Help-seeking preferences in the area of mild cognitive impairment: comparing family physicians and the lay public | Clinical interventions in aging, 9, 613 |
